# Prognostic and predictive role of YKL-40 in anal squamous cell carcinoma: a serological and tissue-based analysis in a multicentric cohort

**DOI:** 10.3389/fmed.2024.1372195

**Published:** 2024-07-09

**Authors:** Alessandro Gambella, Rebecca Senetta, Enrico Costantino Falco, Alessia Andrea Ricci, Luca Mangherini, Cristian Tampieri, Jessica Fissore, Giulia Orlando, Tilde Manetta, Giulio Mengozzi, Massimiliano Mistrangelo, Luca Bertero, Paola Cassoni

**Affiliations:** ^1^Pathology Unit, Department of Medical Sciences, University of Turin, Turin, Italy; ^2^Pathology Unit, Department of Oncology, University of Turin, Turin, Italy; ^3^Pathology Unit, Città della Salute e della Scienza University Hospital, Turin, Italy; ^4^Department of Laboratory Medicine, Città della Salute e della Scienza University Hospital, Turin, Italy; ^5^Department of Surgery, Città della Salute e della Scienza University Hospital, Turin, Italy

**Keywords:** radiochemotherapy, nigro protocol, immunohistochemistry, serum biomarkers, overall survival, response to treatment, YKL-40

## Abstract

**Introduction:**

Anal squamous cell carcinoma (ASC) is a rare gastrointestinal malignancy showing an increased incidence over the past decades. YKL-40 is an immune modulator and pro-angiogenetic factor that showed a promising prognostic and predictive potential in several malignancies, but limited data are available for ASC. This study aims to provide an extensive evaluation of the prognostic and predictive role of YKL-40 in a multicenter cohort of ASC patients.

**Methods:**

We retrospectively retrieved 72 consecutive cases of ASC diagnosed between February 2011 and March 2021. Both serum and tissue protein expression of YKL-40 were assessed, the latter in ASC tumor cells and peritumor immune cells.

**Results:**

Increased YKL-40 serum levels at the time of diagnosis were associated with older age (*p* = 0.035), presence of cardiovascular/metabolic comorbidities (*p* = 0.007), and death for any cause (*p* = 0.011). In addition, high serum levels of YKL-40 were associated with a poor prognosis (HR: 2.82, 95% CI: 1.01–7.84; *p* = 0.047). Protein expression of YKL-40 in ASC tumor cells was significantly associated with low tumor grade (*p* = 0.031), while the increased expression in peritumor immune cells was associated with a worse response of patients to chemoradiotherapy (*p* = 0.007). However, YKL-40 protein expression in ASC tumor cells or peritumor immune cells did not significantly impact patient overall survival.

**Discussion:**

In conclusion, YKL-40 resulted a relevant prognostic (serum level) and predictive (tissue protein expression in peritumor immune cells) biomarker and can considerably improve ASC patient clinical management.

## Introduction

Anal squamous carcinoma (ASC) is a rare gastrointestinal malignancy that represents 2–4% of all colorectal cancer but nearly 90% of all anal cancer. ASC incidence gradually raised in the past decades due to the concurrent increase of human papillomavirus (HPV) and human immunodeficiency virus (HIV) infection rates (1–3). Chronic HPV infection is found in 80–85% of ASC patients and represents one of the most relevant risk factors for ASC development (4, 5), but it is unnecessary for ASC development, and HPV-unrelated ASC is a recognized entity with worse prognosis (6).

After the seminal data published by Nigro et al. (7), ASC patients are mainly treated with radiochemotherapy (CRT) (8–10) that allows sphincter function preservation and achieves complete disease remission in approximately 80% of patients and a 5-year overall survival rate of 60–80%. Unfortunately, almost 10–20% of cases are non-responsive to CRT, and 30% present local disease recurrence, thus requiring salvage surgery (abdominoperineal resection), which substantially affects patients’ quality of life (11–15). Innovative target-therapy approaches, including anti-Epidermal Growth Factor Receptor (EGFR; Cetuximab) and anti-Programmed Cell Death 1 (PD-1) immune checkpoint inhibitors (Nivolumab, Pembrolizumab, and Avelumab), showed promising results but more extensive validations are required (16–18). Based on these considerations, the early identification of ASC patients responding to CRT is crucial, but there is still a critical lack of prognostic and predictive biomarkers, ultimately making the clinical management of non-responsive and recurring ASC challenging.

YKL-40, also known as Chitinase-3-like protein-1 (CHI3L1), is a 40 kDa extracellular matrix glycoprotein encoded by the *CHI3L1* gene located on chromosome 1 (19, 20). YKL-40 is secreted by several cell types, including immune cells and tumor cells, and its expression has been studied in several non-neoplastic (21–28) and neoplastic diseases (29, 30). In the tumor microenvironment, YKL-40 is primarily secreted by tumor-associated macrophages, lymphocytes, and tumor cells (20, 31–33) and is involved in extracellular matrix remodeling, neo-angiogenesis, and immune microenvironment modulation towards an inhibited anti-tumor response (20, 31–33). As a consequence, increased serum levels of YKL-40 have been associated with poor response to treatment and worse prognosis (33–42).

Based on these considerations, we sought to evaluate the serological and tissue protein expression of YKL-40 and assess its prognostic and predictive potential in a multi-institutional series of ASCs.

## Materials and methods

### Data collection and patient clinical management

This retrospective study evaluated the serological levels and tissue protein expression of YKL-40 in a multi-institutional consecutive series of 72 ASCs. ASCs cases were diagnosed between February 2011 and March 2021 and collected by combining the cohorts of all three enrolling institutions (AOU Città della Salute e della Scienza Hospital, Giovanni Bosco Hospital, and Humanitas Gradenigo Hospital, all located in Turin, Italy) and treated according to published guidelines (Supplementary methods) (8–10). Post-treatment follow-up visits were scheduled as follows: every 3–4 months within the initial 3 years after treatment, every 6 months in the following 2 years (four to 5 years after treatment), and every year afterward. Patients with suspected residual or recurrent ASC were first evaluated with digital anorectal examination, anoscopy, and imaging techniques [computerized tomography (CT), positron emission tomography (PET), or magnetic resonance (MRI)] and then confirmed with tissue biopsy. Patients with residual or recurrent ASC were treated with salvage surgery (9, 10).

Patients data were collected and pseudonymized before any analysis was performed (Supplementary Table S1). This study was approved by the Research Ethics Committee of the University of Turin (approval number DSM-ChBU03/2020) following the Helsinki Declaration of 1964 and later versions.

### Immunohistochemical (IHC) stains and related scoring systems

For each case, original slides were reviewed to confirm the diagnosis, assess tissue adequacy, and select a representative formalin-fixed paraffin-embedded (FFPE) tissue block. From each FFPE block, two 3-μm-thick sections were cut to perform p16 (Ventana Medical Systems, Arizona, United States; clone:E6H4; catalog number:06680011001) and YKL-40 (Abcam; Rabbit polyclonal; catalog number:ab180569) IHC stains. p16 and YKL-40 IHC stains were centralized and performed at the Pathology Unit of the AOU Città della Salute e della Scienza Hospital using the automated immunostainer BenchMark XT AutoStainer^®^ (Ventana Medical Systems), as reported (43–45). All immunohistochemical stains (p16 and YKL-40) were evaluated by a pathologist (E.F.) without knowledge of the clinical data, including the oncological outcomes.

The p16 IHC stain was scored using standardized criteria (45). In particular, p16 IHC expression in ≥1% of viable ASC tumor cell nuclei were considered positive, while cases with <1% of positive tumor cell nuclei or with cytoplasmic staining only were considered negative.

As no standardized scoring system for YKL-40 was available, we developed scoring system criteria that mirrored evidence available in the literature. Specifically, we evaluated the percentage of positive viable tumor cells and the related staining intensity and combined them into a four-tier score (Supplementary Table S2), including Score 0 (<1% of positive tumor cells regardless of the staining intensity), Score 1 (weak expression in 1–30% of tumor cells or moderate expression in 1–10% of tumor cells), Score 2 (weak expression in >30% of tumor cells, moderate expression in 10–30% of tumor cells, or strong expression in 1–10% of tumor cells), and Score 3 (moderate expression in >30% of tumor cells or strong expression in >10% of tumor cells). This approach (i.e., combining the percentage of positive tumor cells and the intensity of staining) is commonly used for both experimental (46) and clinically validated prognostic/predictive biomarkers (47, 48).

Peritumor microenvironment immune cells (lymphocytes and macrophages) were also evaluated for YKL-40 expression. The staining intensity resulted homogeneous and, therefore, only the rate of positive immune cells was reported, stratifying ASC cases in low (IC-YKL^LOW^; less than 10% of positive immune cells in the peritumor microenvironment) versus high (IC-YKL^HIGH^; more than 10% of positive immune cells in the peritumor microenvironment). This approach has been used for other biomarkers modulating the immune microenvironment (49).

**Figure 1:**
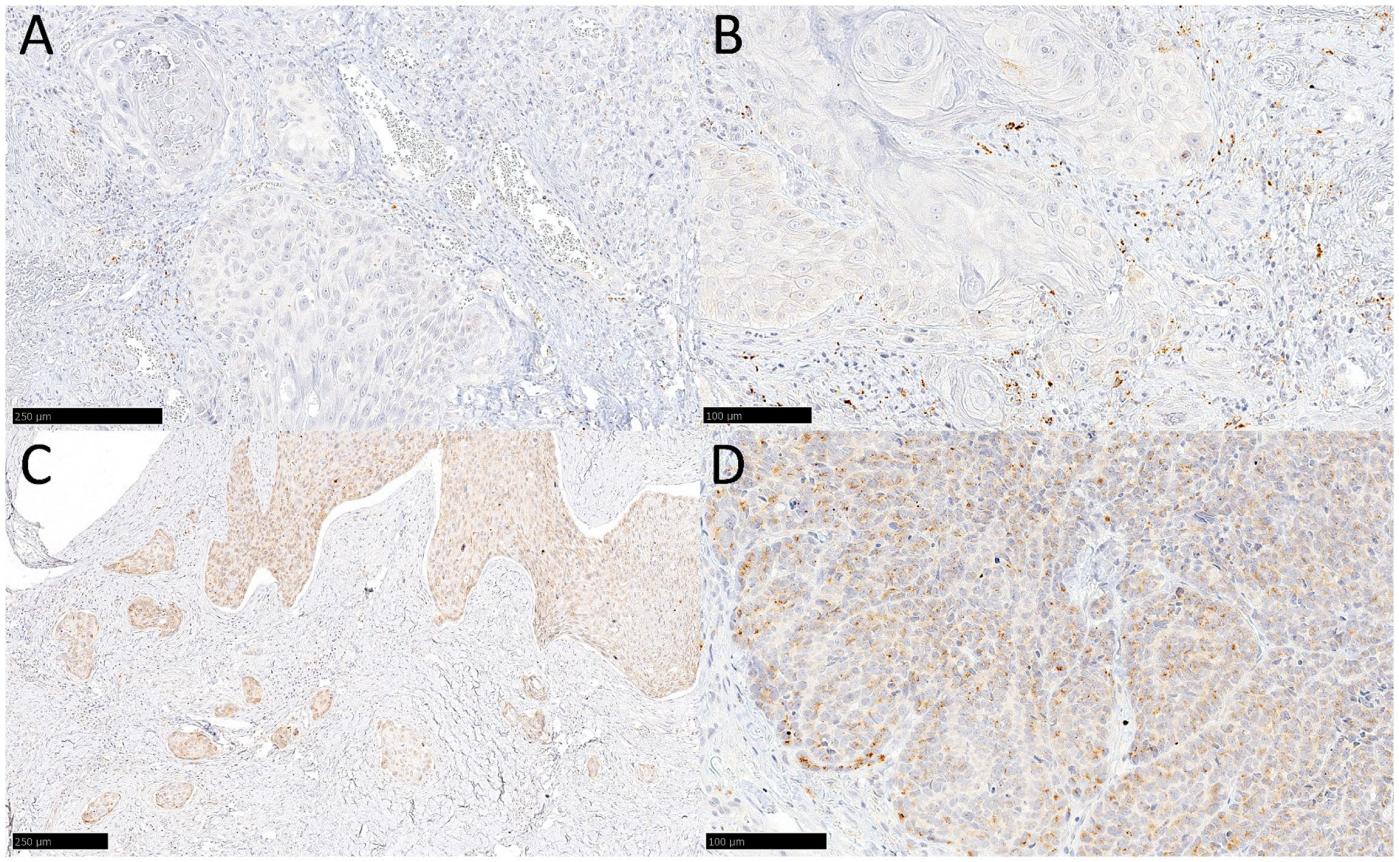
Representative images of YKL-40 protein expression stain. **(A)** Score 0 in tumor cell and IC-YKLLOW. **(B)** Score 0 in tumor cell and IC-YKLHIGH. **(C,D)** Score 3 in tumor cell and IC-YKLLOW at low- **(C)** and high-power **(D)**.

Representative images of the YKL-40 IHC staining are reported in Figure 1.

### YKL-40 assessment in serological samples

Serological samples were collected at the time of diagnosis and during follow-up visits and then centralized to the Clinical Biochemistry Laboratory of the AOU Città della Salute e della Scienza Hospital for YKL-40 quantification. Serum levels of YKL-40 were determined by MicroVue YKL-40 enzyme immunoassay (Quidel, Santa Clara, CA) using streptavidin-coated microplate wells, a biotinylated monoclonal murine antibody against human YKL-40 and an alkaline phosphatase–labeled polyclonal rabbit antibody against human YKL-40. Bound enzyme activity is detected with *p*-nitrophenyl phosphate as substrate. The detection limit of the method is 15.6 ng/mL with a dynamic range up to 300 ng/mL; within-run and between-run precision, as determined by assaying up to 22 serum samples with mean concentrations of 5.8 ng/mL, 177.8 ng/mL and 262.9 ng/mL in 6 different runs, yielded coefficients of variation (CV) <5.8 and < 7.0%, respectively.

### Statistical analysis

Statistical analyses were performed with the Stata 16.0 statistical software (StataCorp, College Station, TX, United States), applying proper tests for parametric (Student t-test) and non-parametric (Pearson chi-square test, Wilcoxon sum rank test) variables and using Bonferroni correction for multiple groups correlations. The follow-up time was reported as the median value and interquartile range (IQR). Overall Survival (OS) was calculated from diagnosis to death, censoring cases at the last follow-up date for lost patients. Survival curves were estimated with the Kaplan–Meier method and compared by the log-rank test. The impact of confounders was explored by the univariable Cox proportional hazard model, including clinical/pathological features as covariates. The proportional hazard assumption test was performed using the scaled Schoenfeld residuals based on the Cox proportional hazard model. All tests were two-sided and statistical analyses considered significant if *p*-value <0.05.

## Results

### Clinicopathological features

Considering the low incidence rate of ASCs, the first part of the study aimed to analyze the clinicopathological features of our series and confirm their pertinence with the data available in the literature (50–52).

In our cohort, the median age was 66 years (IQR:28–85 years), and most patients were female (68.0%, 49/72 cases). Most cases were stage III (54.9%, 39/71) and stage II (33.8%, 24/71) at the time of diagnosis. Accordingly, 57 ASCs patients (79.2%) were treated with CRT and 11 patients (15.3%) underwent local excision of the lesion. Four patients (5.5%) were treated only with radiotherapy due to their clinical condition. The median follow-up time was 5.8 years (IQR:3.1–7.4 years). Most patients (75.4%, 49/65) showed a complete response to treatment, whereas 16 patients (24.6%) presented a partial response and required subsequent salvage surgery. Overall, 24 cases (33.3%) underwent sentinel lymph node biopsy (SNLB) and four of these (16.7%) presented nodal metastasis. During our follow-up period, 19 patients (26.4%) incurred death for any cause. The 5-year overall survival (OS) was 77.5% (Supplementary Figure S1). Demographic, clinical, histopathological, and follow-up data of our cohort are detailed in Supplementary Table S1.

ASCs are slightly more frequent in the adult female population (50–52), as confirmed in our cohort. Based on this consideration, we were interested in evaluating whether any clinicopathological data retrieved was significantly associated with ASCs patient’s sex. Remarkably, well-differentiated (G1) ASC cases were more frequent in the male subgroup compared to females (*p* = 0.016), while CRT treatment was less frequent in the male subgroup compared to females (*p* = 0.018). No additional clinicopathological features were significantly associated with the patient’s sex (Supplementary Table S3).

It is well-known that ASC is related to HPV infection (4, 5). Thus, to further evaluate the adequacy of our cohort, we assessed the protein expression of p16^INK4a^ (p16) in the primary lesions as a surrogate of HPV infection status (53–57). The p16 IHC stain was available for assessment in 67 cases, and most cases (92.5%, 62/67) were positive. Remarkably, p16 expression was significantly associated with the patient’s sex (*p* = 0.011) and response to treatment (*p* = 0.003). Specifically, p16 negative ASC cases were more frequent in the male (20.0%, 4/20) compared to the female (2.1%, 1/47) subgroup and in the non-responsive (26.7%, 4/15) compared to the responsive (2.2%, 1/45) subgroup. No additional clinicopathological features were significantly associated with p16 expression (Supplementary Table S4).

Overall, our cohort of ASCs patients presented data in line with the current literature, specifically confirming the higher incidence in the female population, the frequent p16 expression/HPV infection, and the overall good response of ASC patients to CRT.

### Serological levels of YKL-40 and association with clinicopathological features

To assess the relevance of YKL-40 as a serological biomarker in the ASC setting, we first quantified its serological levels in the ASC cohort and evaluated the association with patients’ clinicopathological features. The median value of serological levels of YKL-40 at the time of diagnosis was 75.6 ng/mL (IQR: 0.7–314.4 ng/mL). We used the median value to stratify ASC patients in cases with high (≥75 ng/mL) versus low (<75 ng/mL) YKL-40 serological levels, and, adopting this stratification, we observed a correlation between high levels and older age at diagnosis (*p* = 0.035), cardiovascular and metabolic comorbidities (*p* = 0.007), and an increased rate of death for any cause (*p* = 0.011) (Table 1).

**Table 1:** Distribution of YKL-40 serum levels according to clinicopathological features.

	Serum YKL-40 < 75 ng/mL (*n* = 37)	Serum YKL-40 ≥ 75 ng/mL (*n* = 35)	*p*-value
Demographical data			
Sex			0.358
Female	27	22	
Male	10	13	
Age (year), median (IQR)	60 (28–81)	69 (43–85)	0.035
Comorbidity			0.007
None	20	8	
Cardiovascular and metabolic	17	27	
Histopathological data and clinical stage			
Tumor grade			0.756
G1	1	2	
G2	11	10	
G3	14	11	
Basaloid features			0.871
No	24	22	
Yes	10	10	
Clinical stage			0.118
Stage I	4	3	
Stage II	8	16	
Stage III	24	15	
Stage IV	0	1	
Therapeutic management and follow-up			
Treatment			0.635
CRT	34	31	
Other	3	4	
Toxicity			0.179
None	1	0	
Minimal	7	6	
Mild	14	10	
Moderate	2	8	
Severe	3	1	
Treatment response			0.347
Complete response	24	25	
No/Partial	10	6	
Sentinel lymph node biopsy			1.000
Negative	10	10	
Positive	2	2	
Disease relapse or recurrence			0.845
No	30	29	
Yes	7	6	
Survival			0.011
No	5	14	
Yes	32	21	

Overall, these findings suggest that YKL-40 serological levels may have a prognostic role for ASC patients.

### YKL-40 in ASC tissue samples: pattern of expression and association with clinicopathological characteristics

Following the analysis of serological YKL-40 levels, we were interested in assessing the tissue protein expression. As YKL-40 can be secreted by both tumor cells and immune cells to regulate the peritumor microenvironment (29, 58), we evaluated both populations separately and subsequently the related significant associations. First, we assessed YKL-40 IHC expression in ASC tumor cells, which was available in 59 cases. According to our scoring system, most ASC cases presented a score 1 (37.3%, 22/59), followed by score 0 (32.2%, 19/59), score 2 (18.6%, 11/59), and score 3 (11.9%, 7/59). The only clinicopathological feature significantly associated with YKL-40 expression in ASC tumor cell was the tumor grade (*p* = 0.031). Specifically, well and moderately differentiated lesions (G1-G2) presented a higher score of YKL-40 (Supplementary Table S5).

Regarding YKL-40 expression in the peritumor immune microenvironment, we evaluated the staining pattern in the macrophages and lymphocytes surrounding ASCs tumor cells. Overall, most cases (75.4%, 36/57) were IC-YKL^LOW^ (<10% of positive immune cells). The expression of YKL-40 in the immune cells was significantly associated with patient response to treatment (*p* = 0.007). In particular, most of the non-responsive ASC patients were IC-YKL^HIGH^ (70%, 7/10). No additional clinicopathological features were significantly associated with YKL-40 expression in the immune cells of the peritumor microenvironment (Supplementary Table S6). Notably, tissue protein expression of YKL-40 in tumor and immune cells were not significantly related (*p* = 0.066; Supplementary Table S7).

This data suggested that the protein expression of YKL-40 in the peritumor immune compartment may play a relevant role as a predictive biomarker for ASC response to CRT.

### Survival analysis and prognostic role of YKL-40

We first evaluated the impact of ASC clinicopathological features on patient overall survival (OS). As expected, complete response to treatment (HR: 0.11, 95% CI: 0.04–0.32; *p* < 0.001) resulted protective for ASC patients, while advanced clinical stage (HR: 3.34, 95% CI: 1.28–8.74; *p* = 0.014) and the presence of residual/recurrent disease during follow-up (HR: 7.03, 95% CI: 2.83–17.4; *p* < 0.001) were associated with a poor prognosis.

**Figure 2:**
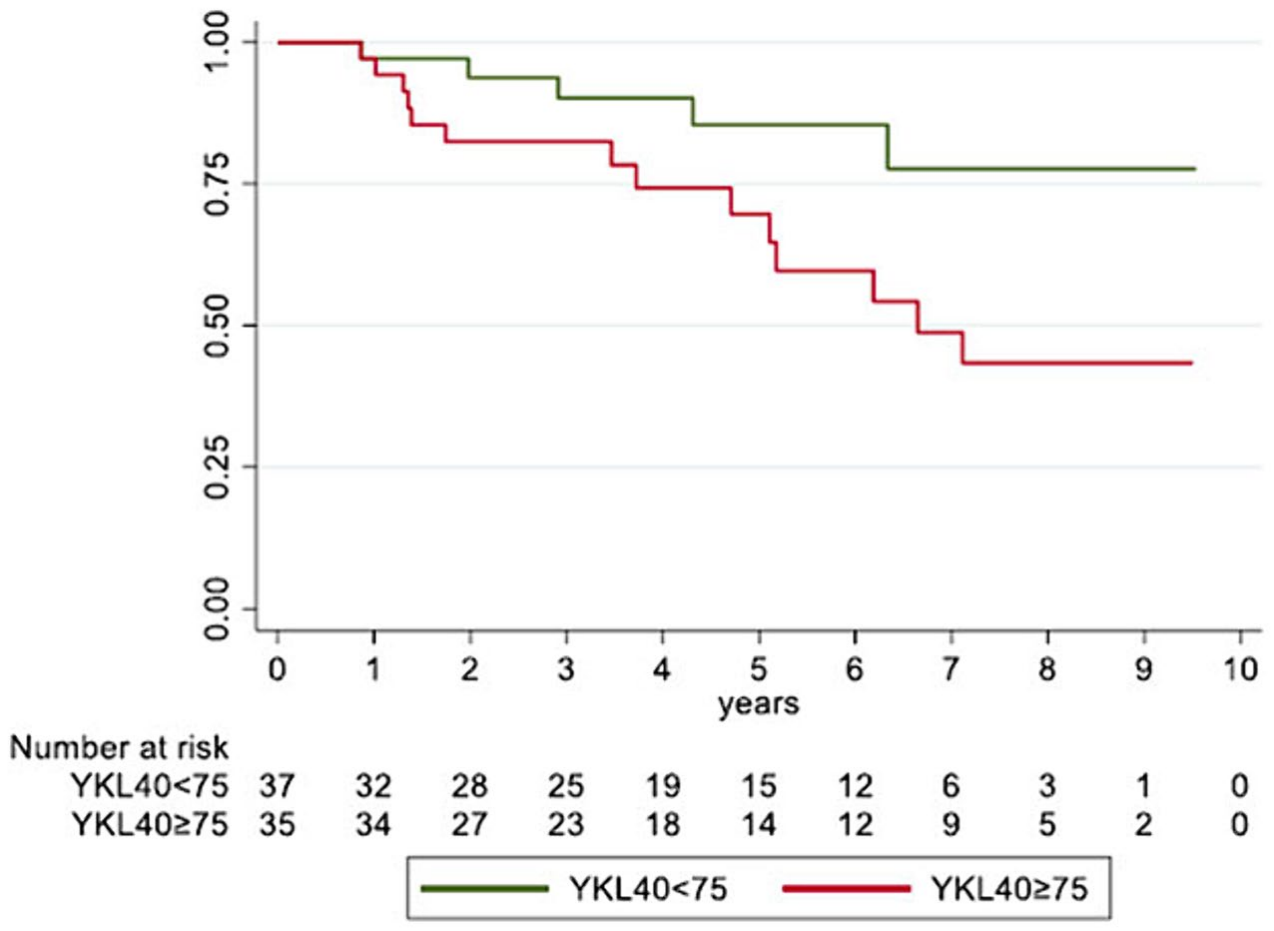
Kaplan-Meyer curve showing the impact of YKL-40 serological levels on patient overall survival.

Then, we focused on the role of YKL-40 expression. Only the serological levels showed a significant impact on patient prognosis (Figure 2). In particular, ASC patients with high serological levels of YKL-40 (≥75 ng/mL) were associated with a worse 5-year survival rate (70.2%) compared to ASC patients with low YKL-40 serum level (85.3%; *p* = 0.038). Furthermore, high serological levels of YKL-40 resulted a significant risk factor for poor prognosis (HR: 2.82, 95% CI: 1.01–7.84; *p* = 0.047). Protein expression of YKL-40 in ASC tumor cells and peritumor immune cells did not show a significant impact on patient survival (Table 2).

**Table 2:** Association between clinicopathological features and patient prognosis (overall survival).

Variables		HR	95% IC	*p*
Sex				
	Male (reference)	1		
	Female	1.80	0.72–4.50	0.206
Age at diagnosis	Linear	1.01	0.96–1.07	0.536
Basaloid features (histopathology)				
	Yes (reference)	1		
	No	1.41	0.54–3.63	0.482
YKL-40 serum levels				
	<75 ng/mL (reference)	1		
	≥75 ng/mL	2.82	1.01–7.84	0.047
YKL-40 tumor cells				
	Score 0 (reference)	1		
	Score 1	1.35	0.43–4.27	0.609
	Score 2	2.10	0.49–9.03	0.317
	Score 3	1.83	0-inf	1.000
YKL-40 immune cells				
	Low (reference)	1		
	High	2.91	0.88–9.63	0.079
Disease relapse or recurrence				
	No (reference)	1		
	Yes	7.03	2.83–17.4	<0.001
Response to treatment				
	None/partial (reference)	1		
	Complete	0.11	0.04–0.32	<0.001
Tumor grade				
	G1 + G2 (reference)	1		
	G3	1.10	0.41–2.98	0.841
Clinical stage	Linear	3.34	1.28–8.74	0.014
p16 expression				
	Negative (reference)	1		
	Positive	0.69	0.15–3.01	0.624
Sentinel Lymph Node Biopsy (SLNB)				
	Negative (reference)	1		
	Positive	3.10	0.50–19.2	0.223

This data confirmed the putative prognostic potential of YKL-40 serum levels.

### Serum YKL-40 and patient follow-up: worth testing?

Considering the impact of serum YKL-40 on ASC patients’ prognosis, we were interested to evaluate its role in the post-treatment follow-up. First, we analyzed and compared the diagnostic yield of all the techniques performed at the first follow-up visit, when 12 of 46 patients (26.1%) presented clinical signs of suspected residual/recurrent disease. Using the histopathological examination/tissue biopsy as the gold standard to confirm residual/recurrent disease, we observed that clinical signs/symptoms (*p* < 0.001), PET (*p* < 0.001), and MRI (*p* < 0.001), but not serum level of YKL-40 (*p* = 0.679; Table 3) were associated with disease recurrence on tissue biopsy.

**Table 3:** Association between histopathological diagnosis of residual/recurrent ASC and the other diagnostic techniques performed at the first follow-up visit.

		Tissue biopsy		*p*-value
		No disease (*n* = 35)	Residual/recurrent ASC (*n* = 7)	
Clinical signs and symptoms				<0.001
	Absent	33	0	
	Present	2	7	
PET				<0.001
	Negative	23	0	
	Positive	6	7	
MRI				<0.001
	Negative	8	1	
	Positive	0	3	
Serum YKL-40				0.679
	Median (IQR)	74.1 (3–332.5)	156.6 (46.7–332.5)	

Then, we were interested in evaluating the temporal trend of serum YKL-40 during the follow-up period. For this longitudinal analysis, we considered only ASC patients with three or more consecutive evaluation of serum YKL-40 (*n* = 17). During the follow-up period, three of the 17 patients (17.6%) incurred death for any cause and the two of them who died due to ASC progression presented high serological levels of YKL-40 (≥75 ng/mL) in all measurements. Notably, the cause of death of the patient with low levels of YKL-40 was not related to ASC. Among the 14 patients alive at the end of the follow-up period, three patients showed high levels of YKL-40 in all measurement but two of them presented cardiovascular and metabolic comorbidities. Two additional patients presented high levels of YKL-40 at the initial assessment, but the value decreased to low levels in subsequent follow-up visits. In the subgroup of nine patients that were alive at the end of the follow-up period and constantly presented low serological levels of YKL-40, only two patients presented cardiovascular and metabolic comorbidities.

Despite the small sample size that hinders a more granular characterization, this analysis of follow-up visits additionally supported the use of serum YKL-40 as a prognostic biomarker for patient overall survival.

## Discussion

In this study, we demonstrated that YKL-40 can represent an innovative biomarker with potential clinical implications for ASC patients management: YKL-40 high serum level identified ASC patients with worse survival and can be used as a prognostic biomarker for patient overall survival, while YKL-40 increased protein expression in the peritumor immune cells characterized ASC patient non-responsive to CRT and can serve as a predictive biomarker.

The prognostic role of YKL-40 has been proved in several tumor setting (20, 29, 31–33, 38, 39, 41, 58), but, remarkably, few data are available for ASC (34, 35, 59). In our cohort, high serum level of YKL-40 was related to an increased rate of death (*p* = 0.011) and resulted a risk factor for ASC patient OS (HR: 2.82, 95% CI: 1.01–7.84; *p* = 0.047), as confirmed by the worse 5-year survival rate of this subgroup of patients (*p* = 0.038). These findings are novel in the ASC setting but in line with data reported in head and neck and cervical squamous cell carcinoma, which presents several pathobiological and clinical similarities with ASCs (60, 61). In particular, high serum YKL-40 identifies a subgroup of patients with head and neck cancer squamous cell carcinoma characterized by worse OS (HR = 1.55, *p* < 0.0001) and relapse-free survival (HR = 2.75, *p* = 0.01) (60). Similar data were observed for cervical cancer, where a high serum level of YKL-40 was associated with worse OS (HR = 1.78, *p* = 0.0010) and relapse-free survival (HR = 1.87, *p* = 0.0016).

Studies evaluating the tissue protein expression of YKL-40 and its impact on patients’ prognosis reported an heterogenous panorama with contrasting data based on the malignancy type (62–65). In the ASC setting, our group previously demonstrated that increased YKL-40 protein expression in ASC tumor cells was related to p53 overexpression/mutation (59), nodal involvement (34) and a worse patient prognosis in terms of OS and disease-free survival (59). In this study, we further characterized YKL-40 tissue protein expression focusing on both the ASC tumor cells and the peritumor immune cells. Remarkably, we observed that the increased expression of YKL-40 in the peritumor immune cells, and not in the tumor cells, was associated with a poor response to CRT (*p* = 0.007) and can be used as a predictive biomarker. This evidence has never been reported in ASC, but was already observed in other malignancies, including CNS tumors and rectal adenocarcinoma (35, 36, 41). In particular, high levels of YKL-40 and c-Met expression identified a subgroup of patients with rectal adenocarcinoma characterized by a poor response to neoadjuvant CRT (35). Based on these data and considering the role played by YKL-40 in modulating the tissue microenvironment (20, 29, 31, 32, 37), it can be hypothesized that YKL-40 production by the peritumor immune cells can dampen the therapeutic effect of CRT. It would be interesting to further address the role of YKL-40 on the peritumor immune microenvironment and ASC response to treatment, especially by evaluating the spatial and longitudinal changes of YKL-40 immune cell protein expression in post-treatment tumor samples.

As a marker associated with the overall immune response, YKL-40 deserves a more granular analysis beyond the oncological field. As an example, YKL-40 can be upregulated by proinflammatory cytokines, including tumor necrosis factor-α, which is a potential therapeutic target for patients affected by rheumatoid arthritis (RA). Interestingly, RA clinical progression was related to YKL-40 level, with RA patients presenting both high levels of serum YKL-40 and increased expression of YKL-40 in synovial cells (66). Based on this evidence and considerations, YKL-40 can be considered a promising diagnostic and therapeutic biomarker of RA. Furthermore, YKL-40 is physiologically expressed in activated microglia and astrocytes, making it a potential diagnostic biomarker for neurological disorders as hypothesized for patients with Alzheimer’s disease (67). Overall, while further studies are needed to fully understand the clinical significance and mechanisms of action of YKL-40 in ASC alone, its potential as a multifaceted diagnostic, prognostic, and predictive biomarker makes it a promising candidate in several neoplastic and non-neoplastic settings.

Combining the advantages of tumor biomarker blood testing (i.e., relatively simple, non- invasive, and cost-effective) with a more specific tissue-tethered assessment allowed us to provide more representative and clinically-useful data regarding the role of YKL-40 in ASC patient management. Still, some limitations are worth mentioning. Despite the multicentric nature, our study is limited by the relatively small sample size that hinders a more granular analysis, especially for patients’ follow-up longitudinal analysis. Indeed, a larger sample size would allow us to (a) explore the effect of confounding variables on YKL-40 prognostic and predictive potentials, including the role of comorbidities, such as autoimmune and cardiovascular diseases and synchronous/metachronous malignancies, and (b) evaluate more detailed and sophisticated scoring systems for YKL-40 tissue expression (i.e., unbiased artificial intelligence-based protein expression assessment). Eventually, our study can benefit from validation in additional cohorts of ASCs, and could be further enriched by the evaluation of YKL-40 expression in pre-cancerous lesions of ASC.

In conclusion, our study produced innovative evidence for YKL-40 use as a prognostic (serum level) and predictive (tissue protein expression in peritumor immune cells) biomarker of ASC. Considering the recent development of targeted treatment against YKL-40 (68, 69), the use of YKL-40 could acquire further predictive relevance in the ASC setting.

## Data availability statement

The raw data supporting the conclusions of this article will be made available by the authors, without undue reservation.

## Ethics statement

The studies involving humans were approved by Research Ethics Committee of the University of Turin (approval number DSM-ChBU03/2020). The studies were conducted in accordance with the local legislation and institutional requirements. The ethics committee/institutional review board waived the requirement of written informed consent for participation from the participants or the participants' legal guardians/next of kin due to the retrospective nature of the research protocol and considering that it had no impact on patients' care.

## Author contributions

AG: Visualization, Writing – original draft, Writing – review & editing. RS: Conceptualization, Data curation, Methodology, Validation, Visualization, Writing – original draft, Writing – review & editing. EF: Data curation, Formal analysis, Methodology, Writing – original draft, Writing – review & editing. AR: Data curation, Formal analysis, Visualization, Writing – original draft, Writing – review & editing. LM: Data curation, Formal analysis, Visualization, Writing – original draft, Writing – review & editing. CT: Data curation, Formal analysis, Visualization, Writing – original draft, Writing – review & editing. JF: Data curation, Formal analysis, Methodology, Investigation, Writing – original draft, Writing – review & editing. GO: Data curation, Formal analysis, Visualization, Writing – original draft, Writing – review & editing. TM: Writing – original draft, Writing – review & editing, Data curation, Formal Analysis, Methodology, Visualization. GM: Data curation, Visualization, Writing – original draft, Writing – review & editing. MM: Data curation, Visualization, Writing – original draft, Writing – review & editing. LB: Conceptualization, Supervision, Validation, Writing – original draft, Writing – review & editing. PC: Conceptualization, Supervision, Validation, Writing – original draft, Writing – review & editing.

## References

[1] Bushara O Krogh K Weinberg SE Finkelman BS Sun L Liao J. Human immunodeficiency virus infection promotes human papillomavirus-mediated anal squamous carcinogenesis: an immunologic and pathobiologic review. Pathobiology. (2022) 89:1–12. doi: 10.1159/000518758, PMID: 34535611

[2] Casadei Gardini A Capelli L Ulivi P Giannini M Freier E Tamberi S. KRAS, BRAF and PIK3CA status in squamous cell anal carcinoma (SCAC). PLoS One. (2014) 9:e92071. doi: 10.1371/journal.pone.0092071, PMID: 24642661 PMC3958420

[3] Spehner L Boustani J Cabel L Doyen J Vienot A Borg C. Present and future research on anal squamous cell carcinoma. Cancers. (2021) 13:3895. doi: 10.3390/cancers13153895, PMID: 34359795 PMC8345786

[4] Park IU Introcaso C Dunne EF. Human papillomavirus and genital warts: a review of the evidence for the 2015 Centers for Disease Control and Prevention sexually transmitted diseases treatment guidelines. Clin Infect Dis. (2015) 61:S849–55. doi: 10.1093/cid/civ813 26602622

[5] Assarzadegan N Brooks E Voltaggio L. HPV-driven anal neoplasia: review and recent developments. Pathology. (2022) 54:184–94. doi: 10.1016/j.pathol.2021.07.003, PMID: 34645567

[6] Glynne-Jones R Saleem W Harrison M Mawdsley S Hall M. Background and current treatment of squamous cell carcinoma of the anus. Oncol Ther. (2016) 4:135–72. doi: 10.1007/s40487-016-0024-0, PMID: 28261646 PMC5315080

[7] Nigro ND Vaitkevicius VK Considine B Jr. Combined therapy for cancer of the anal canal: a preliminary report. Dis Colon Rectum. (1974) 17:354–6. doi: 10.1007/BF02586980 4830803

[8] Flam M John M Pajak TF Petrelli N Myerson R Doggett S. Role of mitomycin in combination with fluorouracil and radiotherapy, and of salvage chemoradiation in the definitive nonsurgical treatment of epidermoid carcinoma of the anal canal: results of a phase III randomized intergroup study. J Clin Oncol. (1996) 14:2527–39. doi: 10.1200/JCO.1996.14.9.2527, PMID: 8823332

[9] Glynne-Jones R Nilsson PJ Aschele C Goh V Peiffert D Cervantes A. Anal cancer: ESMO-ESSO-ESTRO clinical practice guidelines for diagnosis, treatment and follow-up. Radiother Oncol. (2014) 111:330–9. doi: 10.1016/j.radonc.2014.04.013, PMID: 24947004

[10] Glynne-Jones R Nilsson PJ Aschele C Goh V Peiffert D Cervantes A. Anal cancer: ESMO-ESSO-ESTRO clinical practice guidelines for diagnosis, treatment and follow-up. Eur J Surg Oncol. (2014) 40:1165–76. doi: 10.1016/j.ejso.2014.07.030, PMID: 25239441

[11] Pessia B Romano L Giuliani A Lazzarin G Carlei F Schietroma M. Squamous cell anal cancer: management and therapeutic options. Ann Med Surg. (2020) 55:36–46. doi: 10.1016/j.amsu.2020.04.016, PMID: 32461801 PMC7240186

[12] Morton M Melnitchouk N Bleday R. Squamous cell carcinoma of the anal canal. Curr Probl Cancer. (2018) 42:486–92. doi: 10.1016/j.currproblcancer.2018.11.001 30497849

[13] Nelson VM Benson AB 3rd. Epidemiology of Anal Canal Cancer. Surg Oncol Clin N Am. (2017) 26:9–15. doi: 10.1016/j.soc.2016.07.001 27889039

[14] Johnson LG Madeleine MM Newcomer LM Schwartz SM Daling JR. Anal cancer incidence and survival: the surveillance, epidemiology, and end results experience, 1973-2000. Cancer. (2004) 101:281–8. doi: 10.1002/cncr.20364, PMID: 15241824

[15] Clark MA Hartley A Geh JI. Cancer of the anal canal. Lancet Oncol. (2004) 5:149–57. doi: 10.1016/S1470-2045(04)01410-X 15003197

[16] Lukan N Ströbel P Willer A Kripp M Dinter D Mai S. Cetuximab-based treatment of metastatic anal cancer: correlation of response with KRAS mutational status. Oncology. (2009) 77:293–9. doi: 10.1159/000259615, PMID: 19923868

[17] Bamba T Suda T Nakano M Terashima T Umezu H. Pathologically complete response for unresectable stage IV rectal cancer using systemic chemotherapy with panitumumab - a case report. Gan To Kagaku Ryoho. (2012) 39:311–5. https://www.ncbi.nlm.nih.gov/pubmed/22333651 PMID: 22333651

[18] Barmettler H Komminoth P Schmid M Duerr D. Efficacy of Cetuximab in combination with FOLFIRI in a patient with KRAS wild-type metastatic anal Cancer. Case Rep Oncol. (2012) 5:428–33. doi: 10.1159/000341371, PMID: 22949905 PMC3432997

[19] Volck B Price PA Johansen JS Sørensen O Benfield TL Nielsen HJ. YKL-40, a mammalian member of the chitinase family, is a matrix protein of specific granules in human neutrophils. Proc Assoc Am Physicians. (1998) 110:351–60. PMID: 9686683

[20] Shao R Hamel K Petersen L Cao QJ Arenas RB Bigelow C. YKL-40, a secreted glycoprotein, promotes tumor angiogenesis. Oncogene. (2009) 28:4456–68. doi: 10.1038/onc.2009.292, PMID: 19767768 PMC2795793

[21] Seol HJ Lee ES Jung SE Jeong NH Lim JE Park SH. Serum levels of YKL-40 and interleukin-18 and their relationship to disease severity in patients with preeclampsia. J Reprod Immunol. (2009) 79:183–7. doi: 10.1016/j.jri.2008.10.003, PMID: 19200605

[22] Rathcke CN Vestergaard H. YKL-40--an emerging biomarker in cardiovascular disease and diabetes. Cardiovasc Diabetol. (2009) 8:61. doi: 10.1186/1475-2840-8-61, PMID: 19930630 PMC2789050

[23] Hattori N Oda S Sadahiro T Nakamura M Abe R Shinozaki K. YKL-40 identified by proteomic analysis as a biomarker of sepsis. Shock. (2009) 32:393–400. doi: 10.1097/SHK.0b013e31819e2c0c, PMID: 19197227

[24] Zheng JL Lu L Hu J Zhang RY Zhang Q Chen QJ. Increased serum YKL-40 and C-reactive protein levels are associated with angiographic lesion progression in patients with coronary artery disease. Atherosclerosis. (2010) 210:590–5. doi: 10.1016/j.atherosclerosis.2009.12.016, PMID: 20056225

[25] Harvey S Weisman M O'Dell J Scott T Krusemeier M Visor J. Chondrex: new marker of joint disease. Clin Chem. (1998) 44:509–16. doi: 10.1093/clinchem/44.3.509, PMID: 9510855

[26] Furuhashi K Suda T Nakamura Y Inui N Hashimoto D Miwa S. Increased expression of YKL-40, a chitinase-like protein, in serum and lung of patients with idiopathic pulmonary fibrosis. Respir Med. (2010) 104:1204–10. doi: 10.1016/j.rmed.2010.02.026 20347285

[27] Berres ML Papen S Pauels K Schmitz P Zaldivar MM Hellerbrand C. A functional variation in CHI3L1 is associated with severity of liver fibrosis and YKL-40 serum levels in chronic hepatitis C infection. J Hepatol. (2009) 50:370–6. doi: 10.1016/j.jhep.2008.09.016, PMID: 19070929

[28] Bara I Ozier A Girodet PO Carvalho G Cattiaux J Begueret H. Role of YKL-40 in bronchial smooth muscle remodeling in asthma. Am J Respir Crit Care Med. (2012) 185:715–22. doi: 10.1164/rccm.201105-0915OC, PMID: 22281830

[29] Schultz NA Johansen JS. YKL-40-a protein in the field of translational medicine: a role as a biomarker in Cancer patients? Cancers. (2010) 2:1453–91. doi: 10.3390/cancers2031453, PMID: 24281168 PMC3837317

[30] Zhao T Su Z Li Y Zhang X You Q. Chitinase-3 like-protein-1 function and its role in diseases. Signal Transduct Target Ther. (2020) 5:201. doi: 10.1038/s41392-020-00303-7, PMID: 32929074 PMC7490424

[31] Larionova I Tuguzbaeva G Ponomaryova A Stakheyeva M Cherdyntseva N Pavlov V. Tumor-associated macrophages in human breast, colorectal, lung Ovarian Prostate Cancers. Front Oncol. (2020) 10:566511. doi: 10.3389/fonc.2020.566511 33194645 PMC7642726

[32] Pouyafar A Heydarabad MZ Mahboob S Mokhtarzadeh A Rahbarghazi R. Angiogenic potential of YKL-40 in the dynamics of tumor niche. Biomed Pharmacother. (2018) 100:478–85. doi: 10.1016/j.biopha.2018.02.050, PMID: 29477911

[33] Francescone RA Scully S Faibish M Taylor SL Oh D Moral L. Role of YKL-40 in the angiogenesis, radioresistance, and progression of glioblastoma. J Biol Chem. (2011) 286:15332–43. doi: 10.1074/jbc.M110.212514, PMID: 21385870 PMC3083166

[34] Mistrangelo M Senetta R Racca P Castellano I Chiusa L Bellò M. A novel biomarker-based analysis reliably predicts nodal metastases in anal carcinoma: preliminary evidence of therapeutic impact. Color Dis. (2013) 15:1382–91. doi: 10.1111/codi.12289, PMID: 23692332

[35] Senetta R Duregon E Sonetto C Spadi R Mistrangelo M Racca P. YKL-40/c-met expression in rectal cancer biopsies predicts tumor regression following neoadjuvant chemoradiotherapy: a multi-institutional study. PLoS One. (2015) 10:e0123759. doi: 10.1371/journal.pone.0123759, PMID: 25875173 PMC4398550

[36] Senetta R Mellai M Manini C Castellano I Bertero L Pittaro A. Mesenchymal/radioresistant traits in granular astrocytomas: evidence from a combined clinical and molecular approach. Histopathology. (2016) 69:329–37. doi: 10.1111/his.12944, PMID: 26845757

[37] Johansen JS Schultz NA Jensen BV. Plasma YKL-40: a potential new cancer biomarker? Future Oncol. (2009) 5:1065–82. doi: 10.2217/fon.09.66, PMID: 19792974

[38] Høgdall E Johansen JS Kjaer SK Price PA Christensen L Blaakaer J. High plasma YKL-40 level in patients with ovarian cancer stage III is related to shorter survival. Oncol Rep. (2003) 10:1535–8. doi: 10.3892/or.10.5.1535, PMID: 12883737

[39] Pelloski CE Mahajan A Maor M Chang EL Woo S Gilbert M. YKL-40 expression is associated with poorer response to radiation and shorter overall survival in glioblastoma. Clin Cancer Res. (2005) 11:3326–34. doi: 10.1158/1078-0432.CCR-04-1765, PMID: 15867231

[40] Nutt CL Betensky RA Brower MA Batchelor TT Louis DN Stemmer-Rachamimov AO. YKL-40 is a differential diagnostic marker for histologic subtypes of high-grade gliomas. Clin Cancer Res. (2005) 11:2258–64. doi: 10.1158/1078-0432.CCR-04-1601, PMID: 15788675

[41] Qin G Li X Chen Z Liao G Su Y Chen Y. Prognostic value of YKL-40 in patients with glioblastoma: a systematic review and Meta-analysis. Mol Neurobiol. (2017) 54:3264–70. doi: 10.1007/s12035-016-9878-2, PMID: 27090900

[42] Johansen JS Bojesen SE Tybjaerg-Hansen A Mylin AK Price PA Nordestgaard BG. Plasma YKL-40 and total and disease-specific mortality in the general population. Clin Chem. (2010) 56:1580–91. doi: 10.1373/clinchem.2010.146530, PMID: 20798353

[43] Borella F Cosma S Ferraioli D Ray-Coquard I Chopin N Meeus P. Clinical and histopathological predictors of recurrence in uterine smooth muscle tumor of uncertain malignant potential (STUMP): a multicenter retrospective cohort study of tertiary centers. Ann Surg Oncol. (2022) 29:8302–14. doi: 10.1245/s10434-022-12353-y, PMID: 35976464 PMC9640445

[44] Bragoni A Gambella A Pigozzi S Grigolini M Fiocca R Mastracci L. Quality control in diagnostic immunohistochemistry: integrated on-slide positive controls. Histochem Cell Biol. (2017) 148:569–73. doi: 10.1007/s00418-017-1596-y, PMID: 28714056

[45] Darragh TM Colgan TJ Cox JT Heller DS Henry MR Luff RD. The lower Anogenital squamous terminology standardization project for HPV-associated lesions: background and consensus recommendations from the College of American Pathologists and the American Society for Colposcopy and Cervical Pathology. Arch Pathol Lab Med. (2012) 136:1266–97. doi: 10.5858/arpa.LGT200570, PMID: 22742517

[46] Bertero L Gambella A Barreca A Osella-Abate S Chiusa L Francia di Celle P. Caveolin-1 expression predicts favourable outcome and correlates with PDGFRA mutations in gastrointestinal stromal tumours (GISTs). J Clin Pathol. (2022) 75:825–31. doi: 10.1136/jclinpath-2021-207595, PMID: 34155091

[47] Angerilli V Parente P Campora M Ugolini C Battista S Cassoni P. HER2-low in gastro-oesophageal adenocarcinoma: a real-world pathological perspective. J Clin Pathol. (2023) 76:815–21. doi: 10.1136/jcp-2023-208767, PMID: 37055161

[48] Hofmann M Stoss O Shi D Büttner R van de Vijver M Kim W. Assessment of a HER2 scoring system for gastric cancer: results from a validation study. Histopathology. (2008) 52:797–805. doi: 10.1111/j.1365-2559.2008.03028.x, PMID: 18422971

[49] Ilie M Hofman V Dietel M Soria JC Hofman P. Assessment of the PD-L1 status by immunohistochemistry: challenges and perspectives for therapeutic strategies in lung cancer patients. Virchows Arch. (2016) 468:511–25. doi: 10.1007/s00428-016-1910-4, PMID: 26915032

[50] Osborne MC Maykel J Johnson EK Steele SR. Anal squamous cell carcinoma: an evolution in disease and management. World J Gastroenterol. (2014) 20:13052–9. doi: 10.3748/wjg.v20.i36.13052, PMID: 25278699 PMC4177484

[51] Gondal TA Chaudhary N Bajwa H Rauf A Le D Ahmed S. Anal Cancer: the past, present and future. Curr Oncol. (2023) 30:3232–50. doi: 10.3390/curroncol30030246, PMID: 36975459 PMC10047250

[52] Holliday EB Peddireddy A Morris VK. Prognostic and predictive markers for patients with anal Cancer. J Natl Compr Cancer Netw. (2023) 21:678–84. doi: 10.6004/jnccn.2023.7031, PMID: 37308122

[53] Ju JY Stelow EB. Clinicopathologic features of anal and perianal squamous cell carcinomas and their relationship to human papillomavirus. Am J Surg Pathol. (2019) 43:827–34. doi: 10.1097/PAS.0000000000001247, PMID: 31091204

[54] Hoff PM Coudry R Moniz CM. Pathology of anal Cancer. Surg Oncol Clin N Am. (2017) 26:57–71. doi: 10.1016/j.soc.2016.07.013 27889037

[55] Pirog EC. Immunohistochemistry and in situ hybridization for the diagnosis and classification of squamous lesions of the anogenital region. Semin Diagn Pathol. (2015) 32:409–18. doi: 10.1053/j.semdp.2015.02.015, PMID: 25862555

[56] Serup-Hansen E Linnemann D Skovrider-Ruminski W Hogdall E Geertsen PF Havsteen H. Human papillomavirus genotyping and p16 expression as prognostic factors for patients with American joint committee on Cancer stages I to III carcinoma of the anal canal. J Clin Oncol. (2014) 32:1812–7. doi: 10.1200/JCO.2013.52.3464, PMID: 24821878

[57] Lu DW El-Mofty SK Wang HL. Expression of p16, Rb, and p53 proteins in squamous cell carcinomas of the anorectal region harboring human papillomavirus DNA. Mod Pathol. (2003) 16:692–9. doi: 10.1097/01.MP.0000077417.08371.CE, PMID: 12861066

[58] Szymanska-Chabowska A Juzwiszyn J Jankowska-Polanska B Tanski W Chabowski M. Chitinase 3-like 1, nestin, and Testin proteins as novel biomarkers of potential clinical use in colorectal Cancer: a review. Adv Exp Med Biol. (2020) 1279:1–8. doi: 10.1007/5584_2020_506, PMID: 32170669

[59] Castellano I Mistrangelo M Crudo V Chiusa L Lupo R Ricardi U. YKL-40 expression in anal carcinoma predicts shorter overall and disease-free survival. Histopathology. (2009) 55:238–40. doi: 10.1111/j.1365-2559.2009.03364.x, PMID: 19694834

[60] Roslind A Johansen JS Christensen IJ Kiss K Balslev E Nielsen DL. High serum levels of YKL-40 in patients with squamous cell carcinoma of the head and neck are associated with short survival. Int J Cancer. (2008) 122:857–63. doi: 10.1002/ijc.23152, PMID: 17957792

[61] Roslind A Palle C Johansen JS Christensen IJ Nielsen HJ Mosgaard BJ. Prognostic utility of serum YKL-40 in patients with cervical cancer. Scand J Clin Lab Invest. (2020) 80:687–93. doi: 10.1080/00365513.2020.1846209, PMID: 33186077

[62] Kim SH Das K Noreen S Coffman F Hameed M. Prognostic implications of immunohistochemically detected YKL-40 expression in breast cancer. World J Surg Oncol. (2007) 5:17. doi: 10.1186/1477-7819-5-17, PMID: 17286869 PMC1802867

[63] Wan G Xiang L Sun X Wang X Li H Ge W. Elevated YKL-40 expression is associated with a poor prognosis in breast cancer patients. Oncotarget. (2017) 8:5382–91. doi: 10.18632/oncotarget.14280, PMID: 28036271 PMC5354916

[64] Oh IH Pyo JS Son BK. Prognostic impact of YKL-40 Immunohistochemical expression in patients with colorectal Cancer. Curr Oncol. (2021) 28:3139–49. doi: 10.3390/curroncol28040274, PMID: 34436040 PMC8395453

[65] Høgdall EVS Ringsholt M Høgdall CK Christensen IJ Johansen JS Kjaer SK. YKL-40 tissue expression and plasma levels in patients with ovarian cancer. BMC Cancer. (2009) 9:8. doi: 10.1186/1471-2407-9-8, PMID: 19134206 PMC2645422

[66] Tizaoui K Yang JW Lee KH Kim JH Kim M Yoon S. The role of YKL-40 in the pathogenesis of autoimmune diseases: a comprehensive review. Int J Biol Sci. (2022) 18:3731–46. doi: 10.7150/ijbs.67587, PMID: 35813465 PMC9254466

[67] Mavroudis I Chowdhury R Petridis F Karantali E Chatzikonstantinou S Balmus IM. YKL-40 as a potential biomarker for the differential diagnosis of Alzheimer's disease. Medicina. (2021) 58:60. doi: 10.3390/medicina58010060 35056368 PMC8777884

[68] Faibish M Francescone R Bentley B Yan W Shao R. A YKL-40-neutralizing antibody blocks tumor angiogenesis and progression: a potential therapeutic agent in cancers. Mol Cancer Ther. (2011) 10:742–51. doi: 10.1158/1535-7163.MCT-10-0868, PMID: 21357475 PMC3091949

[69] Libreros S Garcia-Areas R Shibata Y Carrio R Torroella-Kouri M Iragavarapu-Charyulu V. Induction of proinflammatory mediators by CHI3L1 is reduced by chitin treatment: decreased tumor metastasis in a breast cancer model. Int J Cancer. (2012) 131:377–86. doi: 10.1002/ijc.26379, PMID: 21866546 PMC3288379

