# Supplementary material for: Prognostic and predictive role of YKL-40 in anal squamous cell carcinoma: a serological and tissue-based analysis in a multicentric cohort
Source: Front Med (Lausanne). 2024 Jul 9;11:1372195. doi: 10.3389/fmed.2024.1372195 (PMC11263350; doi:10.3389/fmed.2024.1372195)
Supplement: Supplementary file 1 [file Data_Sheet_1.PDF]

Prognostic and predictive role of YKL-40 in anal squamous cell carcinoma: a serological and tissue-based analysis in a multicentric cohort.

Alessandro Gambella[1], Rebecca Senetta[2], Enrico Costantino Falco[3], Alessia Andrea Ricci[1], Luca Mangherini[1], Cristian Tampieri[1], Jessica Fissore[3], Giulia Orlando[2], Tilde Manetta[4], Giulio Mengozzi[1], Massimiliano Mistrangelo[5], Luca Bertero[1], Paola Cassoni[1]

[1] Pathology Unit, Department of Medical Sciences, University of Turin, Turin, Italy
[2] Pathology Unit, Department of Oncology, University of Turin, Turin, Italy
[3] Pathology Unit, Città della Salute e della Scienza University Hospital, Turin, Italy
[4] Department of Laboratory Medicine, Città della Salute e della Scienza University Hospital, Turin, Italy
[5] Department of Surgery, Città della Salute e della Scienza University Hospital, Turin, Italy

Supplementary material

Index

Supplementary Methods	***Page 2***
Supplementary Tables	***Pages 3 - 12***
Supplementary Table 1. Demographic, clinical, histopathological, and follow-up data of the ASC cohort.	*Page 3*
Supplementary Table 2. Immunoreactivity score used to evaluate YKL-40 expression in tumor cells and peritumor immune cells.	*Page 5*
Supplementary Table 3. Clinicopathological features stratified according to patient sex.	*Page 6*
Supplementary table 4. Association between p16 expression and patient clinicopathological features.	*Page 8*
Supplementary Table 5. Association between YKL-40 expression in tumor cells and patient clinicopathological features.	*Page 10*
Supplementary Table 6. Association between YKL-40 expression in peritumor immune cells and patient clinicopathological features.	*Page 12*
Supplementary Table 7. Association between protein expression of YKL-40 in tumor and peritumor immune cells.	*Page 14*
Supplementary Figures	***Page 15***
Supplementary Figure 1. Kaplan-Meyer curve of our cohort of ASC patients.	*Page 15*
Supplementary References	***Page 16***

Supplementary Method

Patients with stage I-III ASC of the anal canal and patients with stage II-III ASC of the anal margin were treated with CRT, thus combining intensity-modulated radiotherapy with 5-Fluorouracyl and mitomycin-C. Patients with stage I ASCs of the anal margin (i.e., cT1 N0 M0) were treated with local excision of the lesion. Post-operative low-dose CRT was performed if surgical margins were positive on histological examination (<1 mm of tumor-free tissue from surgical resection margin).

In selected patients, sentinel lymph node biopsy (SLNB) was performed to assess lymph node status for subsequent inguinal lymphadenectomy[1,2].

Post-treatment follow-up visits were performed to evaluate treatment toxicity and residual or recurrent disease. Treatment toxicity was recorded according to the criteria of the Common Terminology Criteria for Adverse Events (version 4)[3,4].

Supplementary table 1. Demographic, clinical, histopathological, and follow-up data of the ASC cohort.

	ASC cohort (n=72)
Demographical data	
**Sex**	
**Female**	49 (68.0%)
**Male**	23 (32.0%)
**Age (year), median (IQR)**	66 (28-85)
**Comorbidity**	
**No**	28 (38.9%)
**Yes**	44 (61.1%)
**Type of comorbidity**	
**Hypertension and hypercholesterolemia**	34 (77.3%)
**Hypertension, hypercholesterolemia and diabetes**	10 (22.7%)
Histopathological data and clinical stage	
**Tumor grade**	
**G1**	3 (6.1%)
**G2**	21 (42.9%)
**G3**	25 (51.0%)
**Basaloid features**	
**No**	46 (69.7%)
**Yes**	20 (30.3%)
**Clinical stage**	
**Stage I**	7 (9.8%)
**Stage II**	24 (33.8%)
**Stage III**	39 (54.9%)
**Stage IV**	1 (1.4%)
Therapeutic management and follow-up	
**Treatment**	
**CRT**	57 (79.2%)
**Local Excision**	11 (15.3%)
**Other**	4 (5.5%)
**Toxicity**	
**None**	1 (1.6%)
**Minimal**	23 (37.1%)
**Mild**	24 (38.7%)
**Moderate**	10 (16.1%)
**Severe**	4 (6.4%)
**Treatment response**	
**Complete response**	49 (75.4%)
**No/Partial**	16 (24.6%)
**Sentinel lymph node biopsy**	

	**Negative**	20 (83.3%)
	**Positive**	4 (16.7%)
**Follow-up (year), median (IQR)**		5.8 (3.1-7.4)
**5-year overall survival**		77.5%
**Disease relapse or recurrence**		
	**No**	59 (81.9%)
	**Yes**	13 (18.1%)
**Survival**		
	**No**	19 (26.4%)
	**Yes**	53 (73.6%)

Supplementary Table 2. Immunoreactivity score used to evaluate YKL-40 expression in tumor cells and peritumor immune cells.

	Score 0	Score 1	Score 2	Score 3
**Tumor cells**	Any stain intensity in <1% of TCs	Weak expression in ≥1-30% of TCs<br>or<br>Moderate expression in ≥1-10% of TCs	Weak expression in ≥30% of viable cells<br>or<br>Moderate expression in ≥10-30% of TCs<br>or<br>Strong expression in <10% of TCs	Moderate expression in ≥30% of TCs<br>or<br>Strong expression in ≥10% of TCs
**Peritumor immune cells (macrophages and lymphocytes**	**IC-YKL^{LOW}**		**IC-YKL^{HIGH}**	
	<10% of positive immune cells in the peritumor microenvironment		≥10% of positive immune cells in the peritumor microenvironment	

Supplementary Table 3. Clinicopathological features stratified according to patient sex.

	ASC female patients (n=49)	ASC male patients (n=23)	p-value
Demographical data			
**Age (year), median (IQR)**	66 (43-85)	66 (43-85)	0.663
**Comorbidity**			0.418
**No**	20	8	
**Cardiovascular and metabolic**	24	10	
**Diabetes**	5	5	
Histopathological data and clinical stage			
**Tumor grade**			**0.016**
**G1**	0	3	
**G2**	13	8	
**G3**	20	5	
**Basaloid features**			0.230
**No**	30	16	
**Yes**	16	4	
**Clinical stage**			0.352
**Stage I**	3	4	
**Stage II**	16	8	
**Stage III**	29	10	
**Stage IV**	1	0	
Therapeutic management and follow-up			
**Treatment**			**0.018**
**CRT**	47	10	
**Other**	2	5	
**Toxicity**			0.216
**None**	0	1	
**Minimal**	10	13	
**Mild**	19	5	
**Moderate**	6	4	
**Severe**	4	0	
**Treatment response**			0.313
**Complete response**	37	12	
**No/Partial**	10	6	
**SLNB**			1.000
**Negative**	15	5	
**Positive**	3	1	
**Disease relapse or recurrence**			0.920
**No**	40	19	
**Yes**	9	4	

**Survival**			
**No**	11	8	0.268
**Yes**	38	15	

Supplementary Table 4. Association between p16 expression and patient clinicopathological features.

	p16 negative ASCs (n=5)	p16 positive ASCs (n=62)	p-value
Demographical data			
**Sex**			
**Female**	1	46	**0.011**
**Male**	4	16	
**Age (year), median (IQR)**	71 (28-78)	71 (28-78)	0.116
**Comorbidity**			
**None**	1	22	0.648
**Cardiovascular and metabolic**	4	38	
Histopathological data and clinical stage			
**Tumor grade**			
**G1**	0	3	0.313
**G2**	4	17	
**G3**	1	22	
**Basaloid features**			
**No**	5	39	0.126
**Yes**	0	19	
**Clinical stage**			
**Stage I**	0	6	0.702
**Stage II**	1	21	
**Stage III**	4	33	
**Stage IV**	0	1	
Therapeutic management and follow-up			
**Treatment**			
**CRT**	5	55	0.427
**Other**	0	7	
**Toxicity**			
**None**	0	0	0.750
**Minimal**	2	10	
**Mild**	2	21	
**Moderate**	0	9	
**Severe**	0	4	
**Treatment response**			
**Complete response**	1	44	**0.003**
**No/Partial**	4	11	
**SLNB**			
**Negative**	1	6	0.490
**Positive**	0	3	

**Disease relapse or recurrence**			0.575
**No**	5	48	
**Yes**	0	12	
**Survival**			0.491
**No**	2	16	
**Yes**	3	46	

Supplementary Table 5. Association between YKL-40 expression in tumor cells and patient clinicopathological features.

Features		Total (n=59)	YKL-40 expression in ASC tumor cells				p-value
			Score 0 (n=19)	Score 1 (n=22)	Score 2 (n=11)	Score 3 (n=7)	
Demographical data							
**Sex**							
	**Female**	41	11	16	9	5	0.549
	**Male**	18	8	6	2	2	
**Age (year), median (IQR)**		66 (43-85)	66 (52-81)	66 (43-85)	69 (43-78)	64 (52-79)	0.997
Histopathological data and clinical stage							
**Tumor grade**							
	**G1-G2**	20	9	3	6	2	**0.031**
	**G3**	19	4	11	4	0	
**Basaloid features**							
	**No**	36	9	12	10	5	0.212
	**Yes**	19	7	9	1	2	
**p16 expression**							
	**Negative**	4	1	3	0	0	0.389
	**Positive**	55	18	19	11	7	
**YKL-40 serum level**	**Median (IQR)**	70.5 (0.7-314)	84.7 (3-314)	62.4 (0.7-300)	99.7 (25-169)	58.1 (13.6-140)	0.447
**YKL-40 serum level**							
	**<75 ng/mL**	30	8	14	5	3	0.507
	**≥75 ng/mL**	29	11	8	6	4	
**Clinical stage**							
	**Stage I**	6	2	1	2	1	0.530
	**Stage II**	21	9	8	1	3	
	**Stage III**	30	7	2	8	3	
	**Stage IV**	1	0	1	0	0	
Therapeutic management and follow-up							
**Treatment**							
	**CRT**	52	16	22	8	6	0.123
	**Other**	7	3	0	3	1	
**Toxicity**							
	**None-Mild**	33	9	18	3	3	0.154
	**Moderate-Severe**	11	5	2	3	1	

**Sentinel lymph node biopsy**							
	**Negative**	7	4	2	1	0	0.728
	**Positive**	3	1	1	1	0	
**Response to treatment**							
	**Complete**	41	15	14	7	5	0.132
	**No/partial**	11	1	8	1	1	
**Survival**							
	**No**	15	14	15	8	7	0.409
	**Yes**	44	5	7	3	0	

Supplementary Table 6. Association between YKL-40 expression in peritumor immune cells and patient clinicopathological features.

Features		Total (n=57)	YKL-40 expression in immune cells		p-value
			Low (n=36)	High (n=21)	
Demographical data					
**Sex**					
	**Female**	39	25	14	0.828
	**Male**	18	11	7	
**Age (year), median (IQR)**		67 (43-85)	65 (43-79)	71 (43-85)	0.388
Histopathological data and clinical stage					
**Tumor grade**					
	**G1-G2**	20	13	7	0.699
	**G3**	17	10	7	
**Basaloid features**					
	**No**	35	20	15	0.229
	**Yes**	19	14	5	
**p16 expression**					
	**Negative**	4	2	2	0.572
	**Positive**	53	34	19	
**YKL-40 serum level**	**Median (IQR)**	70.5 (0.7-314)	70.8 (0.7-302.7)	69.8 (13.6-314)	0.717
**YKL-40 serum level**					
	**<75 ng/mL**	29	15	14	0.069
	**≥75 ng/mL**	28	21	7	
**Clinical stage**					
	**Stage I**	6	3	3	0.252
	**Stage II**	21	16	5	
	**Stage III**	28	16	12	
	**Stage IV**	1	0	1	
Therapeutic management and follow-up					
**Treatment**					
	**CRT**	50	33	17	0.234
	**Other**	7	3	4	
**Toxicity**					
	**None-Mild**	32	22	10	0.405
	**Moderate-Severe**	11	9	2	
**Response to treatment**					
	**Complete**	40	30	10	**0.007**
	**No/partial**	10	3	7	

**Sentinel lymph node biopsy**					
	**Negative**	7	6	1	0.686
	**Positive**	1	1	0	
**Survival**					
	**No**	14	9	5	0.920
	**Yes**	43	27	16	

Supplementary table 7. Association between protein expression of YKL-40 in tumor cells and peritumor immune cells.

		IC-YKL^{LOW}	IC-YKL^{HIGH}	p-value
YKL-40 in tumor cells	Score 0	14	3	0.066
	Score 1	14	7	
	Score 2	5	5	
	Score 3	2	5	

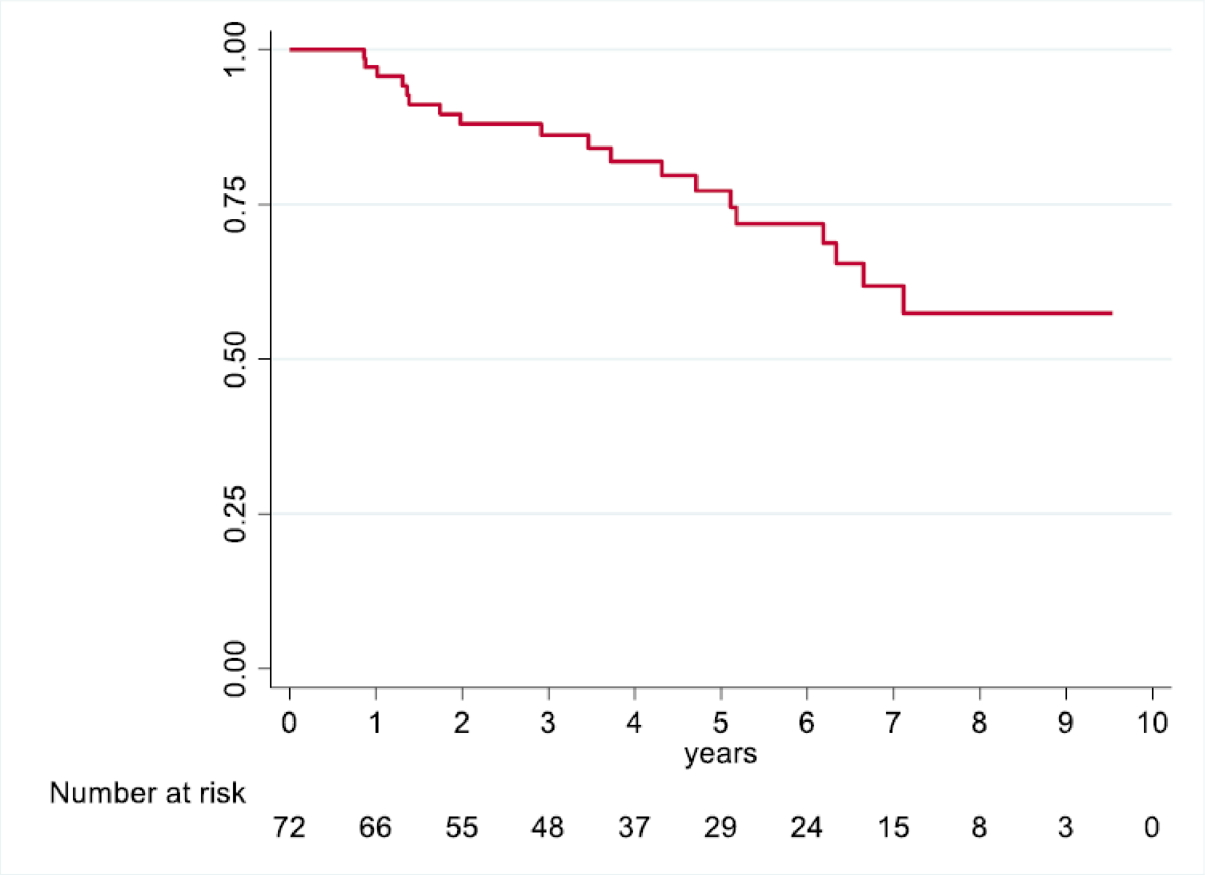

Supplementary Figure 1. Kaplan-Meier curve of our cohort of ASC patients.

References

1. de Jong, J.S., *et al.* Limited value of staging squamous cell carcinoma of the anal margin and canal using the sentinel lymph node procedure: a prospective study with long-term follow-up. *Ann Surg Oncol* **17**, 2656-2662 (2010).
2. Mistrangelo, D.M., *et al.* Value of staging squamous cell carcinoma of the anal margin and canal using the sentinel lymph node procedure: an update of the series and a review of the literature. *Br J Cancer* **108**, 527-532 (2013).
3. Brown, E., *et al.* Acute toxicity and patient-reported outcomes in anal canal cancer: results of a pilot study. *J Med Radiat Sci* **69**, 484-491 (2022).
4. Health, U.D.o. & Human Services %J National Institutes of Health, N.C.I. Common terminology criteria for adverse events (CTCAE) version 4.0. **4**(2009).
